# Genomic characterization of *Streptococcus parasuis*, a close relative of *Streptococcus suis* and also a potential opportunistic zoonotic pathogen

**DOI:** 10.1186/s12864-022-08710-6

**Published:** 2022-06-25

**Authors:** Genglin Guo, Zhuohao Wang, Quan Li, Yanfei Yu, Yubao Li, Zhongming Tan, Wei Zhang

**Affiliations:** 1 College of Veterinary Medicine, Nanjing Agricultural University, Nanjing, 210095 China; 2 Key Lab of Animal Bacteriology, Ministry of Agriculture, Nanjing, 210095 China; 3 OIE Reference Lab for Swine Streptococcosis, Nanjing, 210095 China; 4 MOE Joint International Research Laboratory of Animal Health and Food Safety, Nanjing, China; 5 The Sanya Institute of Nanjing Agricultural University, Sanya, China; 6 College of Veterinary Medicine, Yangzhou University, Yangzhou, China; 7 Key Laboratory of Veterinary Biological Engineering and Technology of Ministry of Agriculture, Institute of Veterinary Medicine, Jiangsu Academy of Agricultural Sciences, Nanjing, China; 8 Agricultural Science and Engineering School, Liaocheng University, Liaocheng, China; 9 NHC Key laboratory of Enteric Pathogenic Microbiology, Jiangsu Provincial Center for Disease Control and Prevention, Nanjing, 210009 China

**Keywords:** *Streptococcus parasuis*, Pan-genome, Capsular polysaccharide, ICE, Pili

## Abstract

*Streptococcus parasuis* (*S. parasuis*) is a close relative of *Streptococcus suis* (*S. suis*), composed of former members of *S. suis* serotypes 20, 22 and 26. *S. parasuis* could infect pigs and cows, and recently, human infection cases have been reported, making *S. parasuis* a potential opportunistic zoonotic pathogen. In this study, we analysed the genomic characteristics of *S. parasuis*, using pan-genome analysis, and compare some phenotypic determinants such as capsular polysaccharide, integrative conjugative elements, CRISPR-Cas system and pili, and predicted the potential virulence genes by associated analysis of the clinical condition of isolated source animals and genotypes. Furthermore, to discuss the relationship with *S. suis*, we compared these characteristics of *S. parasuis* with those of *S. suis*. We found that the characteristics of *S. parasuis* are similar to those of *S. suis*, both of them have “open” pan-genome, their antimicrobial resistance gene profiles are similar and a *srtF* pilus cluster of *S. suis* was identified in *S. parasuis* genome. But *S. parasuis* still have its unique characteristics, two novel pilus clusters are and three different type CRISPR-Cas system were found. Therefore, this study provides novel insights into the interspecific and intraspecific genetic characteristics of *S. parasuis*, which can be useful for further study of this opportunistic pathogen, such as serotyping, diagnostics, vaccine development, and study of the pathogenesis mechanism.

**Supplementary Information:**

The online version contains supplementary material available at 10.1186/s12864-022-08710-6.

## Introduction

The emergence of novel pathogens is considered a major hazard to public health [1]. The genus *Streptococcus* is a highly diverse group comprising more than 100 pathogenic or commensal species. Among them, *Streptococcus pyogenes*, *S. agalactiae* and *S. pneumoniae* are the most common human opportunistic pathogens which commonly colonise the respiratory, digestive and genitourinary tracts [2]. Usually, commensal *Streptococci* intimately colonises with other bacteria species in host tissues. Bacterial genomes are highly plastic, allowing bacteria to rapidly regulate it metabolism in response to new niches and changes in environmental conditions [3]. Exploring the genetic evolution of bacteria, combined with sequencing efforts, has allowed us to better understand the molecular and evolutionary changes and analyse the living patterns of these bacteria, thereby preventing the infection caused by these bacteria.

*Streptococcus suis* (*S. suis*) is a zoonotic pathogen that can infect both humans and swine. Despite the fact that there are more than 50 distinct serotypes have been identified (29 classic serotypes and 26 novel capsular polysaccharide loci [NCL] serotypes), the majority of them do not infect the host and instead masquerade as commensal flora in the upper respiratory tract [4]. *Streptococcus parasuis* (*S. parasuis*) is a close relative of *S. suis*, initially classified as *S. suis* serotypes 20, 22 and 26. In 2015, Nomoto et al. reappraised the taxonomy and named it *S. parasuis* based on average nucleotide identity, 16S ribosomal ribonucleic acid (rRNA), and biochemical features [5]. *S. parasuis* has been isolated from both healthy and diseased pigs and cows and can cause symptoms and diseases similar to *S. suis*, including meningitis, pneumonia, septicemia, endocarditis and arthritis [6, 7]. Recently, two human *S. parasuis* infection cases were reported in China, making *S. parasuis* a potential opportunistic zoonotic pathogen and hazardous to public health [8].

Bacterial genomes can be best described as consisting of core and accessory genomes. The core genome represents genes essential for survival and colonisation. The accessory genome represents a set of genes specific to one isolate, which commonly plays an important role in the evolution of bacterial pathogens. In this study, pan-genome analysis was performed to explore the genome structure of *S. parasuis* and compare it with it closely related, *S. suis*, to discuss the evolutionary differences. To further discuss the genomic characteristics of *S. parasuis*, several components of the genome, such as the capsular polysaccharide (CPS) biosynthesis locus, integrative conjugative elements (ICE), CRISPR-Cas systems and pilus gene cluster, are associated with many important phenotypes such as virulence [9], serotype [10] and antimicrobial resistance (AMR) [11] were extracted from the genome of *S. parasuis*, analysed, and compared with those of *S. suis*. Therefore, this study aims to understand both interspecies and intraspecies genetic characteristics of *S. parasuis* by combining analysis using pan-genome and alignment of phenotype-associated determinants*.*

## Materials and methods

### Genome data of *S. parasuis*

All acquirable genome data of *S. parasuis* were downloaded from the National Centre for Biotechnology Information (NCBI) by searching “*Streptococcus parasuis*” in the “Genome” database, and nine genomes were found. Further, considering that *S. parasuis* was separated from *S. suis* and several genome data were listed in the “*Streptococcus suis*” section, more genome data of *S. parasuis* were obtained by searching the associated published paper in “PubMed” database, and six genomes were found (Table 1). Two raw sequencing datasets, SUT-319 and SUT-328, were assembled using Unicycler [12]. The average nucleotide identity (ANI) and tetranucleotide frequencies (Tetra) of these genomes were calculated using JSpeciesWS to measure the probability of the genomes belonging to the same species [13]. Finally, the assembly quality of the draft genomes used in this study was assessed using QUAST [14] and CheckM [15], and the details are listed in Additional file 1.

**Table 1:** Details of *S. parasuis* genomes used in this study.

Strains	Source	Location	Year	Accession No.	complete?	plasmid	Length	cps type
SUT-380	Healthy pig	Japan	2013	AP024277.1	Y	2	2,109,881	VI
SUT-503	Healthy pig	Japan	2014	AP024280.1	Y	0	2,065,066	XI*
SUT-286	Healthy pig	Japan	2013	AP024276.1	Y	0	2,197,342	IV
SUT-7	Healthy pig	Japan	2012	AP024275.1	Y	0	2,202,836	V
BS27	Patient	China	2018	JAETXU000000000.1	N	/	1,909,795	X
BS26	Patient	China	2018	CP069079.1	Y	0	1,932,292	X
H35	Healthy pig	China	2018	CP076721.1	Y	1	2,186,318	XII*
4253	Healthy cow	Switzerland	2018	SHGT00000000.1	N	/	1,881,656	IX
86–5192	Diseased calf	United States	1980’	ALLG00000000.1	N	/	2,110,166	I
88–1861	Diseased pig	Canada	1980’	ALLW00000000.1	N	/	2,272,254	II
89–4109-1	Diseased pig	/	1980’	ALLL00000000.1	N	/	2,176,728	III
SUT-319	Healthy pig	Japan	/	DRX016753	N	/	2,129,893	VI
SUT-328	Healthy pig	Japan	/	DRX016754	N	/	2,088,627	VI
10–36,905	Healthy *Bos taurus*	United States	2010	WNXH00000000.1	N	/	2,148,541	VII
2843	Healthy pig	China	2014	POIG00000000.1	N	/	2,267,031	VIII

* Typing in this study

### Multi locus sequence typing (MLST)

There is no multi locus sequence typing (MLST) database for *S. parasuis*; however, considering that *S. parasuis* was separated from *S. suis*, we analysed these *S. parasuis* genome data using the *S. suis* database [16]. As a result, all seven housekeeping genes, *aroA*, *cpn60*, *dpr*, *gki*, *mutS*, *recA*, and *thrA*, of *S. suis* could be found in the *S. parasuis* genome. The phylogenetic tree was generated by MEGA X using the neighbour-joining method [17].

### Pan-genome analysis and identification of orthologous

The genome data were re-annotated by Prokka to combine the FASTA and GFF format data [18]. The pan-genome of *S. parasuis* was investigated using Roary, and genomic characteristics were visualised using a roary_plots.py script [19]. The core and accessory genome data generated using Roary were further annotated by eggnog-mapper to identify the orthologous proteins by the cluster of orthologous group (COG) [20].

### Comparison of the capsular polysaccharide (CPS) biosynthesis loci

The first and last *cps* flanking genes of *S. parasuis* from the published *cps* locus were used to screen for their presence in 14 *S. parasuis* genomes [8]. In addition. The potential *cps* locci were aligned using ClustalW [21]. The phylogenetic tree was generated by MEGA X using the neighbour-joining method [17], and visualised by both EasyFig [22] and Mauve [23] to look for the variation. The locations of the potential *cps* locus are listed in Additional file 2.

### Detection of antimicrobial resistance genes (AMR) and integrative conjugative elements (ICE)

AMRG were screened using ResFinder 4.1 [24]. ICE were predicted using ICEfinder [11], the draft genome data were predicted using the FASTA format file, and the complete genome data were predicted using the Genbank format file. All available *S. suis* ICEs in the ICEberg database were downloaded and analysed in this study, and the details are listed in Additional file 3.

### Screen the pili cluster in *S. parasuis* genome

All pili clusters of *S. suis* were searched for in the *S. parasuis* genome using BLASTp. Further, to detect more potential pili clusters in *S. parasuis*, the keywords “sortase”, “pili(n)”, “pilus” were screened in the annotation Genbank files. The reference sequences for the pili clusters of *S. suis* are listed in Additional file 4.

### Prediction of CRISPR-Cas system in *S. parasuis* genome

CRISPR-Cas systems were predicted using CRISPRCasFinder [25], and only completed CRISPR and CRISPR with Cas were counted.

### Prediction of the potential virulence-associated genes by phenotype association study

To identify the potential virulence-associated genes of S. parasuis, the known virulence marker of *S. suis*, capsular polysaccharides (CPS) muramidase-released protein (MRP), suilysin (SLY) and extracellular factor (EF), were scanned in the genomes of S. parasuis using BLASTp. Furthermore, a genotype-phenotype association study was performed. The gene presence and absence data generated by the pan-genome analysis tool Roary were used, and the clinical conditions of the isolation source animals were used as phenotypes. In addition, a genome-wide association study tool, treeWAS, was used to analyse genes related to this phenotype [26, 27]. The potential functions of the predicted genes were annotated by alignment using BLASTp and the Conserved Domain Database (CDD).

## Results

### Characteristics of *S. parasuis*

Fifteen *S. parasuis* genome sequences were downloaded from the public database. First, the species of these isolates were checked at the genomic level. The ANI and Tetra of one isolate, 2843, also named 2674 in a previous study [8] (recognised by the same Genbank number), are highly different from the other 14 *S. parasuis* isolates. Further, we analysed the genome composition and found that 79% genes (1736/2198) of this isolate are unique and the size core-genome has a huge reduction after adding this isolate to analyses (from 1043 to 264) (Additional file 5). Therefore, we believe that this isolate did not belong to *S. parasuis* and was excluded from this study.

*S. parasuis* has a wide range of hosts, and the source of these 14 isolates included healthy or diseased humans, pigs and cows. Human infection cases caused by *S. parasuis* have been reported recently. Considering that pigs and cows are major livestock in most countries and the human-livestock contact is very frequent, and the distribution of *S. parasuis* is also broad, including in Europe, North America and Asia, making this species a potential emerging opportunistic zoonotic pathogen.

The chromosome sizes of these *S. parasuis* isolates range from 1.90 Mb (BS27) to 2.27 Mb (88–1861), with a mean size of 2.10 Mb. Interestingly, we found that the chromosome sizes of the two human isolates are much shorter than that of the mean size. However, colinear analysis using complete genomes of human isolate BS26 and five pig isolates found no chromosome deletion, translocation, or rearrangement of large fragments (Additional file 6). The virulence and host difference may cause by a single gene (cluster) or single nucleotide polymorphism (SNP).

### Subtyping of *S. parasuis* by MLST

**Fig. 1:**
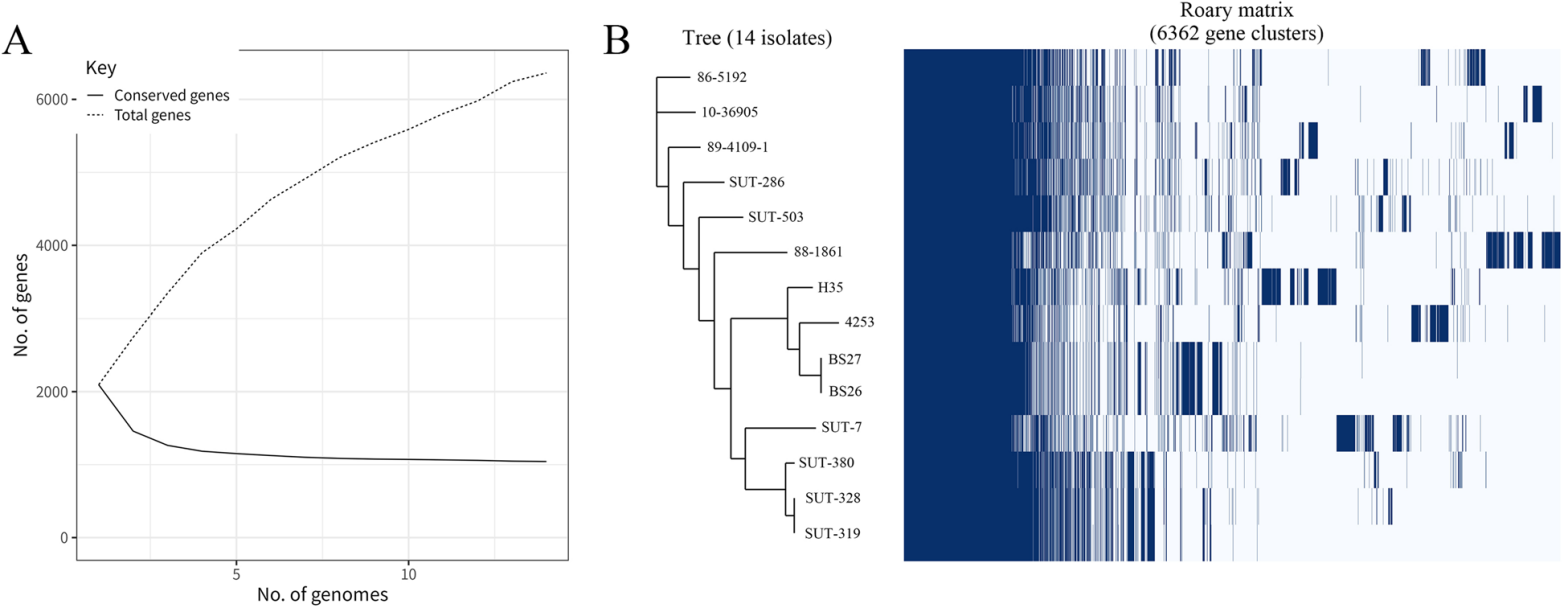
Pan-genome analysis of 14 *S. parasuis* isolates. **A** The number of core or pan genes curve of *S. parasuis* as the increasing number of the genomes; **B** The presence and absence matrix of the 14 *S. parasuis* isolates

Considering that *S. parasuis* is initially belonged to *S. suis*, the *S. suis* MLST database was used, and seven housekeeping genes, *aroA*, *cpn60*, *dpr*, *gki*, *mutS*, *recA*, and *thrA* could also be found in the genome of *S. parasuis*. We found that only BS26 and BS27, SUT-319 and SUT-328 belonged to the same sequence type (ST). The other ten isolates had different MLST profiles, however, every *S. parasuis* isolate shared at least one allele sequence with others, except H35. Apart from that, despite some allele sequences being different, they are still closely linked, such as *thrA*, seven isolates (SUT-380, SUT-286, 86–5192, 89–4109-1, SUT-319, SUT-328, and 10–36,905) matched with allele 127. The other two isolates, SUT-7 and SUT-503, had no match in the MLST database, but the closest match was 127, this phenomenon could also be found in other housekeeping genes. Our findings indicated that these *S. parasuis* isolates were closely related to genetic lineages (Table 2). A phylogenetic tree was built using these seven sequences, combining analyse the core genome alignment, we found that the MLST tree can partial reflect the WGS result, for example, in both phylogenetic trees, BS26, BS27, 4253 and H35 belong to one clade and SUT-380, SUT-319 and SUT-328 belong to one clade (Additional file 7 and Fig. 1B).

**Table 2:** MLST analysis result of *S. parasuis* genomes

Strains	aroA	cpn60	dpr	gki	mutS	recA	thrA	ST
SUT-380	233	454	91	260	344*	279	127	/
SUT-503	194*	128	202*	303*	344*	102	127*	/
SUT-286	233*	124*	84	305*	256	102	127	/
SUT-7	231*	382	269	238	176	279*	127*	/
BS27	297*	83	171	222	417	208	227*	/
BS26	297*	83	171	222	417	208	227*	/
H35	300	84*	173*	83	83	67	228	/
4253	297*	83	214	222*	417*	161*	227*	/
86–5192	264	197	202	302	344*	220	127	/
88–1861	265	197	215	303	176	138	128	946
89–4109-1	265	197	202	220	344	102	127	/
SUT-319	194*	454	91	394	344*	279	127	/
SUT-328	194*	454	91	394	344*	279	127	/
10–36,905	194	197	202	221	256	102	127	1289

* Closest match

### Genomic phylogenetic analysis of *S. parasuis*

To observe the phylogenetic evolutionary relationship of *S. parasuis* at the whole-genome level, a pan-genome analysis was performed. Although the pan-genome of these 14 *S. parasuis* isolates has a total number of 6362 different genes, consisting of 1043 core genes (99% ≤ isolates ≤100%), 1655 shell genes (15% ≤ isolates ≤95%), and 3664 cloud genes (0% ≤ isolates ≤15%) (Additional file 8), the enormous number of cloud genes indicating the genome of each isolate varied considerably. For pan-genome analysis, the numbers of core and pan-genome were calculated every time a new genome was added. As a result, the core-genome curve fit well into a decaying function and the pan-genome curve is a not stabilised asymptotic value with the genome number increased, suggesting that the *S. parasuis* has an “open pan-genome”, the same as *S. suis* (Fig. 1A).

To investigate the phylogenetic relationships among these 15 isolates, a neighbor-joining tree was constructed based on core genome alignment, and a gene presence and absence matrix was built (Fig. 1B). Similar to *S. suis*, some *S. parasuis* isolates belonging to the same *cps* type were grouped in a clade, such as *cps* type X (BS26 and BS27) and VI (SUT-319, SUT-328 and SUT-380).

**Fig. 2:**
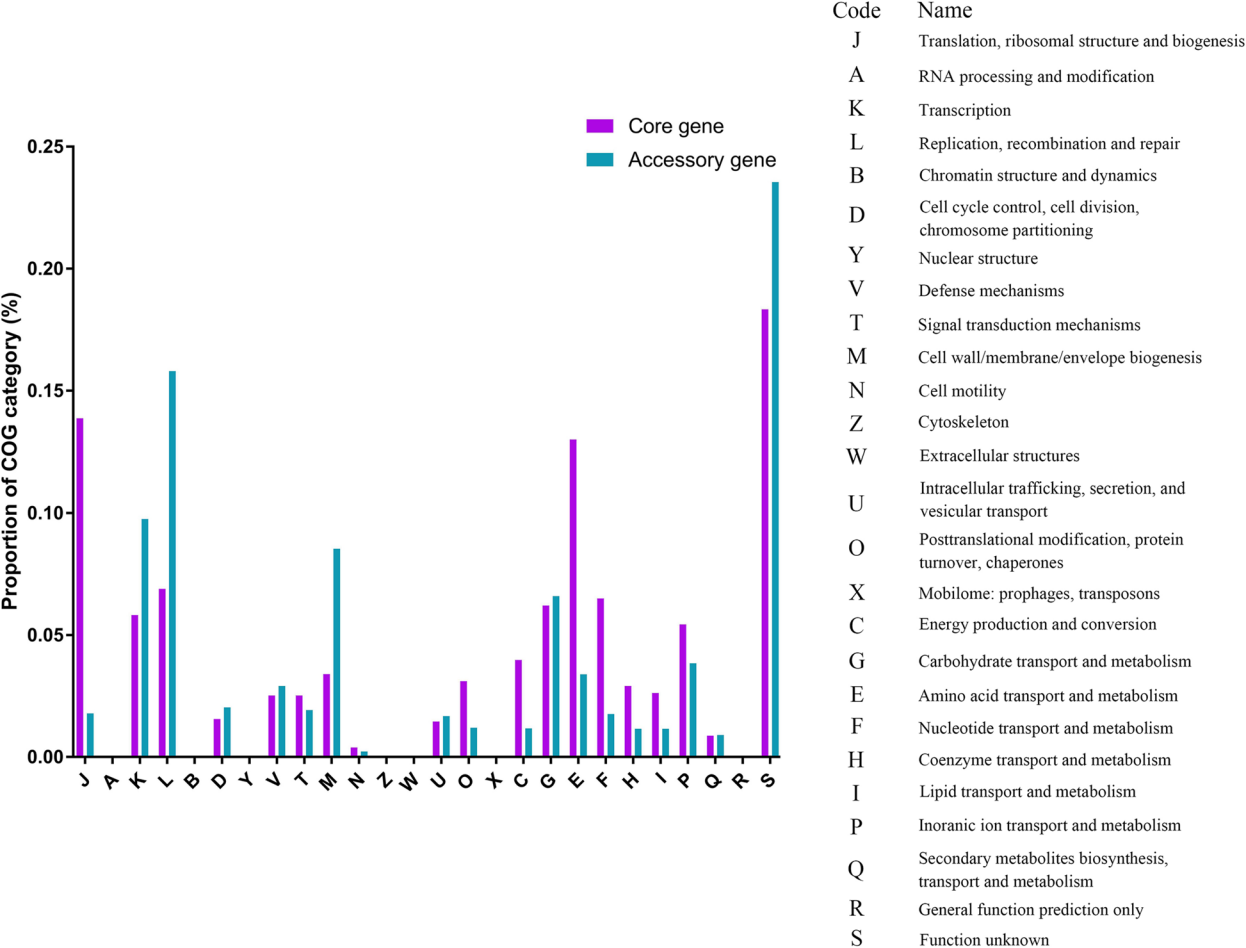
Cluster of orthologous groups (COGs) functional classification of core gene and accessory gene of *S. parasuis*

The coding proteins of all genes of *S. parasuis* were annotated in the Database of Clusters of Orthologous Genes (COGs) [28]. Only assigned COG functional genes were considered. The different function preferences of the core and accessory genomes were analysed. The core genes of *S. parasuis* were more often associated with COG categories J (translation, ribosomal structure and biogenesis), F (Nucleotide transport and metabolism) and E (Amino acid transport and metabolism), whereas accessory genes of *S. parasuis* were more often associated with COG categories L (Replication, recombination and repair), K (Transcription) and M (Cell wall/membrane/envelop biogenesis) (Fig. 2). This finding indicates that the core genes of *S. parasuis* are preferred for basic physiological and biological functions, and that the functions of accessory genes are involved in genetic evolution, adapt to novel environments or treatments.

### Analysis of capsular polysaccharide (CPS) biosynthesis loci of *S. parasuis*

**Fig. 3:**
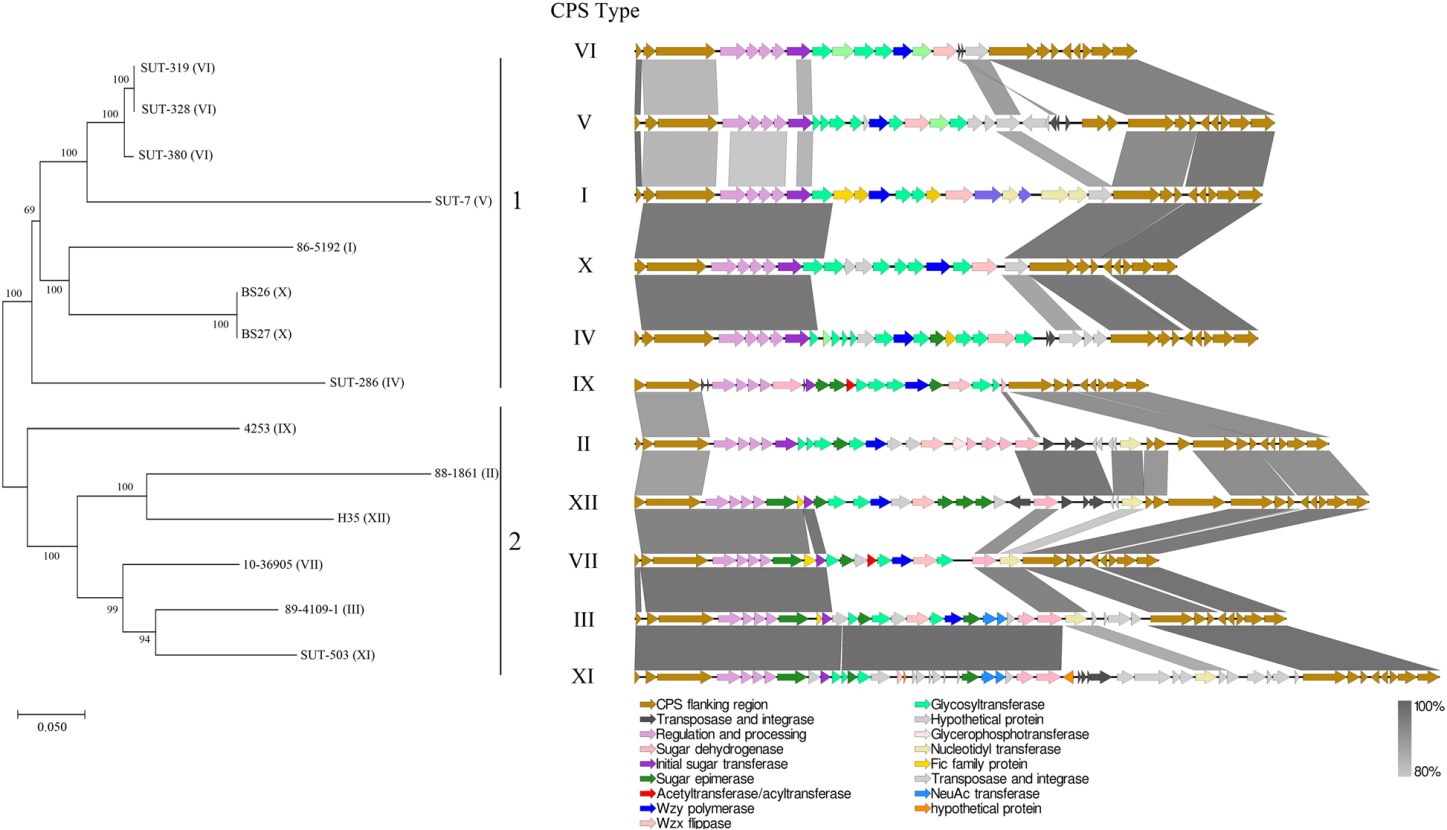
Phylogenetic analysis of capsular polysaccharide (CPS) biosynthesis locus of *S. parasuis*. The *cps* locus in the same clade is colinear aligned using EasyFig

In the present study, to explore the differences in the *S. parasuis cps* locus at the intraspecies level, the *cps* locus of every *S. parasuis* isolates was identified, extracted from the genome, and aligned to investigate the potential crosslink. Eleven different *cps* types were identified in this study (excluding type VIII from isolate 2843, discussed in Section 3.1). The phylogenetic tree based on the entire length of the *cps* locus demonstrated that the *cps* of *S. parasuis* could be separated into two clades (Fig. 3). Furthermore, the gene structure of *S. parasuis* was collinearity visualised (Additional file 9) and aligned within each clade. All *cps* locus of *S. parasuis* shared four highly conserved four *cps* biosynthesis regulation and processing genes, *cpsA*, *cpsB*, *cpsC*, and *cpsD*, and flanking regions. The middle section of the *cps* locus is diverse. Compared with clade 1, the *cps* locus of clade 2 contained more sugar epimerase or dehydrogenase, and less glycosyltransferase, indicating that despite the high intraspecies heterogeneity of the CPS structure, there may be much more difference between different clades and some similarity within the clades, which still needs to be proven by molecular analysis.

### Antimicrobial resistance gene and integrative conjugative elements profiles of *S. parasuis*

AMR is an important public concern. In this study, we scanned the AMRG of *S. parasuis* genome and found the tetracycline resistance gene *tet(O/M)* and aminoglycoside resistance gene *ant(6)-Ia* had high isolation rates (9/14, both of them), followed by erythromycin resistance gene *erm(B*) (8/14) (Table 3).

**Table 3:** Prediction of antimicrobial resistance gene and integrative conjugative element (ICE) in *S. parasuis* genomes.

Strains	number of ICE	Name of ICE	Location	Size (bp)	GC content (%)	resistance genes	Does ICE contain resistance genes?
SUT-380	/					erm(B), ant(6)-Ia, tet(L), tet(O)	/
SUT-503	/					/	/
SUT-286	3	ICESpsSUT-286-1	910,877–1,034,765	123,889	38.81	tet(M), ant(6)-Ia	Y
		ICESpsSUT-286-2	1,759,153–1,810,570	51,418	39.39		
		ICESpsSUT-286-3	1,842,722–1,907,421	64,700	37.77		
SUT-7	/					erm(B), aph(3′)-III, ant(6)-Ia, mef(A)	/
BS27	/					msr(D), mef(A)	/
BS26	/					msr(D), mef(A)	/
H35	2	ICESpsH35–1	1,607,033–1,676,868	69,836	36.52	msr(D), erm(B), ant(6)-Ia, aac(6′)-aph(2″), lsa(E), mdt(A), tet(M), optrA, mef(A), catQ, lnu(B), Cfr(D)	Y
		ICESpsH35–2	2,018,960–2,081,277	62,318	38.99		
4253	/					/	/
86–5192	/					erm(B), tet(O), ant(6)-Ia	/
88–1861	1	ICESps88–1861	ALLW01000055.1 (126,339 - end), ALLW01000056.1, ALLW01000057.1, ALLW01000058.1, ALLW01000059.1,ALLW01000060.1, ALLW01000061.1 (start - 53,947)	106,501	40.22	erm(B), tet(O), ant(6)-Ia	N
89–4109-1	2	ICESps89–4109-1-1	ALLL01000008.1 (18,381 - end), ALLL01000009.1, ALLL01000010.1, ALLL01000011.1, ALLL01000012.1, ALLL01000013.1 (start - 14,394)	161,688	40.74	erm(B), tet(O), ant(6)-Ia, lnu(C)	Y
		ICESps89–4109-1-2	ALLL01000049.1 (459 - end), ALLL01000050.1, ALLL01000051.1, ALLL01000052.1, ALLL01000053.1 (start - 7605)	77,346	38.45		
SUT-319	2	ICESpsSUT-319-1	contig001 (134186–247,211)	113,026	38.95	erm(B), ant(6)-Ia, tet(L), tet(O)	Y
		ICESpsSUT-319-2	contig004 (120,272 - end), contig005 (start - 97,118)	135,071	40.22		
SUT-328	1	ICESpsSUT-328-1	contig003 (61,960 - end), contig004 (start - 20,840)	119,180	39.74	erm(B), ant(6)-Ia, tet(L), tet(O)	Y
10–36,905	/					tet(M)	/

**Fig. 4:**
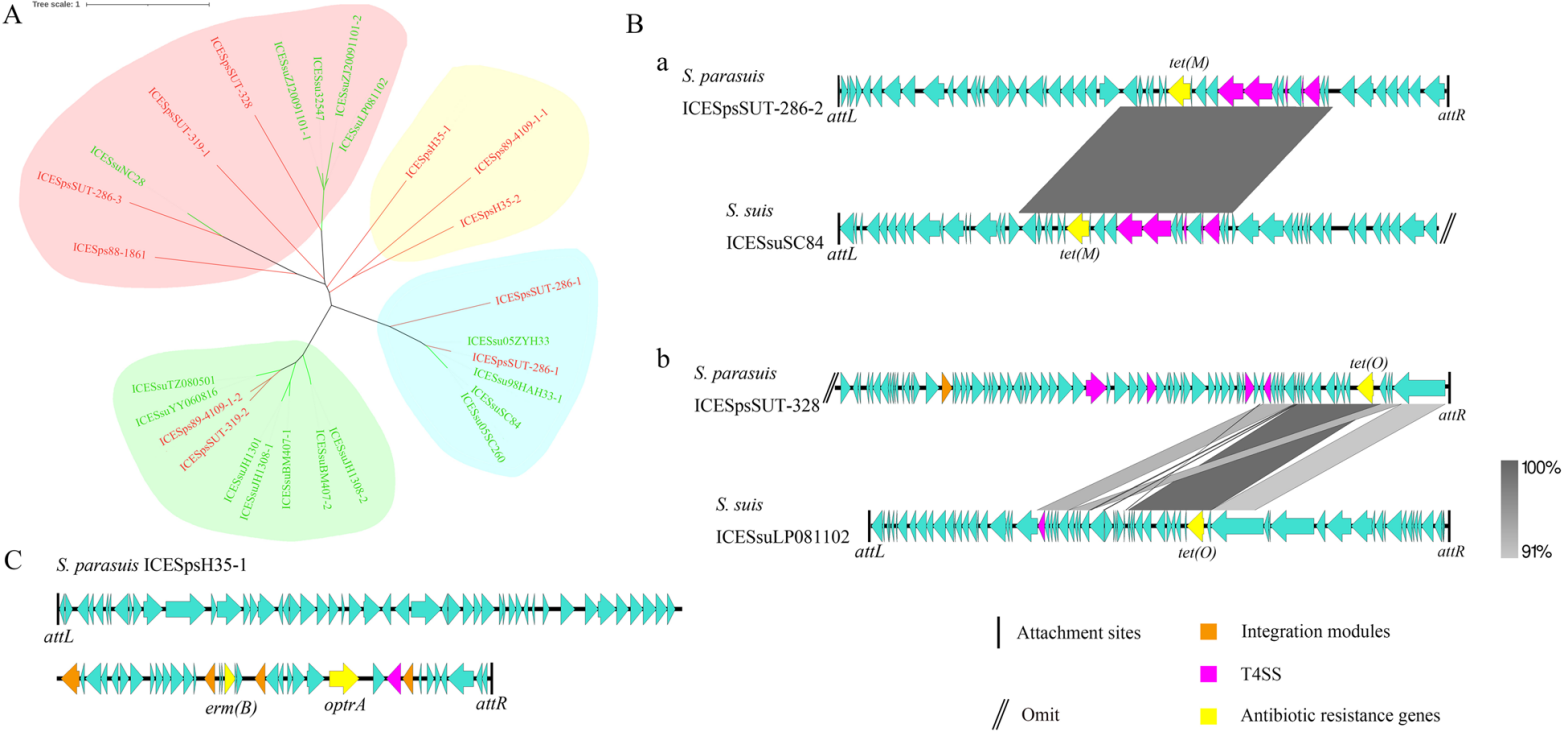
**A** Phylogenetic tree of ICEs identified in *S. parasuis* and *S. suis*, the red branch is ICEs from *S. parasuis* and green branch is *S. suis*; **B** Colinear alignment of ICEs from *S. parasuis* and *S. suis* in the same clade; **C** A schematic map of the genetic structure of ICESpsH35–1

ICE structures of *S. parasuis* were predicted by using ICEfinder, and 11 different ICEs were predicted in 6 isolates. Of these six isolates five had AMRGs located in ICEs (Table 3). Furthermore, to investigate whether there are phylogenetic links between the ICE of *S. parasuis* and *S. suis*, a phylogenetic tree was constructed, and we observed that there are four main clades of these ICEs, and three of them contain ICE from both *S. parasuis* and *S. suis* genomes (Fig. 4A). To observe the connection, two groups of ICE from *S. parasuis* and *S. suis* in the same clade were chosen to perform the analysis, and we can see that there is a large range of similarities in both groups (Fig. 4B). These findings indicate that horizontal gene transfer may have occurred between these two pathogens. In addition, one clade that contains only ICE from *S. parasuis*, indicating that *S. parasuis* may have its own characteristics that differ from *S. suis*. Interestingly, we found that the *optr(A)* gene, previously reported to be co-harbored with *cfr(D)* in *S. parasuis*, is located in an ICE (Fig. 4C).

### Identification and prevalence of putative pilus gene clusters of *S. parasuis*

**Fig. 5:**
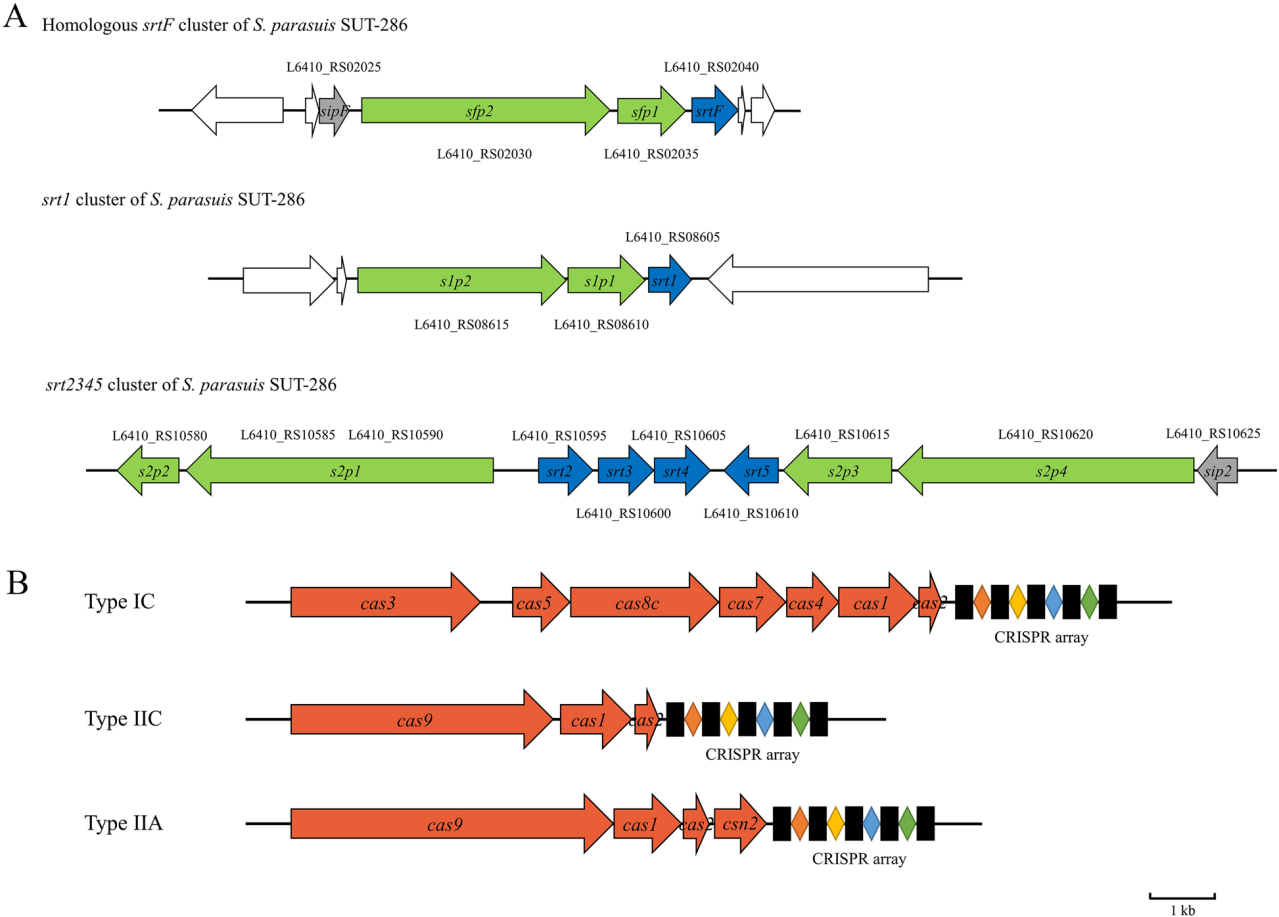
Genomic organization of (**A**) pilus gene clusters in *S. parasuis* and the locus tag in the genome, blue arrows are class C sortase genes, green arrows are pilin subunit coding genes, gray arrows are genes encoding signal peptidase homologues and white arrows are flanking genes; **B** CRISPR-Cas systems. Orange arrows are cas family proteins

We scanned for the presence of homologous gene clusters of pilus gene clusters of *S. suis* in *S. parasuis*, and only a homologous *srtF* cluster was found. However, two novel pilus gene clusters were found in *S. parasuis*, which are not homologous in *S. suis* (Fig. 5A). Considering that Takamatsu et al. named the *S. suis* pilus gene cluster alphabetically, we named these novel pilus gene clusters of *S. parasuis* in Arabic numerical order to avoid confusion: *srt1* and *srt2345* cluster, respectively. It should be noted that the *srt2345* pili cluster contains complicated gene structures and it may not be ae single pili cluster, and we hope that future researchers could determine that; however, it does not affect the analysis in this study.

Based on the presence or absence of pilus gene clusters, *S. parasuis* could be separated into four genotypes (Table 4). Among the three pilus gene clusters, the *srt1* cluster is ubiquitous in every *S. parasuis* genome,.Four isolates are missing the *srtF* cluster, three isolates contain the complete *srt2345* cluster, and one isolate contains partial. In addition, we observed an association between pili genotype and the clinical condition of source animals or *cps* type, no obvious association was found.

**Table 4:** Prediction of pili cluster in *S. parasuis* genomes

Strains	srtA	srtF cluster				srt1 cluster		
		srtF	sfp1	sfp2	sipF	srt1	s1p1	s1p2
10–36,905	GLP18_07430	GLP18_08055	GLP18_08050	GLP18_08045	GLP18_08040	GLP18_01855	GLP18_01860	GLP18_01865
86–5192	SST18_RS0106025	SST18_RS0102600	SST18_RS0102595	SST18_RS0102590	SST18_RS0102585	SST18_RS0100080	SST18_RS1000000111105	SST18_RS0100090
88–1861	SST34_RS0102675					SST34_RS0100095	SST34_RS1000000112435	SST34_RS0100105
89–4109-1	SST23_RS0109685	SST23_RS0103550	SST23_RS0103555	SST23_RS0103560	SST23_RS0103565	SST23_RS0105610	SST23_RS0105615	SST23_RS0105620
4253	EXW74_04430					EXW74_02595	EXW74_02600	EXW74_02605
BS26	JOA01_RS04580	JOA01_RS09350	JOA01_RS09355	JOA01_RS09360	JOA01_RS09365	JOA01_RS07550	JOA01_RS07555	JOA01_RS07560
BS27	JM961_05600	JM961_08150	JM961_08155	JM961_08160	JM961_08165	JM961_02310	JM961_02315	JM961_02320
H35	KQ224_RS01520					KQ224_RS09100	KQ224_RS09095	KQ224_RS09090
SUT-7	SUT007_10200					SUT007_17200	SUT007_17190	SUT007_17200
SUT-286	L6410_RS04875	L6410_RS02040	L6410_RS02035	L6410_RS02030	L6410_RS02025	L6410_RS08605	L6410_RS08605	L6410_RS08615
SUT-319	+*	+	+	+	+	+	+	+
SUT-328	+	+	+	+	+	+	+	+
SUT-380	SUT380_09340	SUT380_03770	SUT380_03760	SUT380_03770	SUT380_03740	SUT380_16370	SUT380_16380	SUT380_16390
SUT-503	SUT503_09340	SUT503_19780	SUT503_19790	SUT503_19800	SUT503_19810	SUT503_16330	SUT503_16340	SUT503_16350

Strains	srtA	srt2345 cluster									Genotype
		srt2	srt3	srt4	srt5	s2p1	s2p2	s2p3	s2p4	sip2	
10–36,905	GLP18_07430	GLP18_05745	GLP18_05740	GLP18_05735	GLP18_05730	GLP18_05750	GLP18_05755	GLP18_05725	GLP18_05720	GLP18_05715	A
86–5192	SST18_RS0106025	SST18_RS0101355	SST18_RS0101360	SST18_RS0101365	SST18_RS0101370	SST18_RS1000000111685, SST18_RS1000000111690	SST18_RS0101345	SST18_RS0101375	SST18_RS1000000111110	SST18_RS0101385	A
88–1861	SST34_RS0102675										B
89–4109-1	SST23_RS0109685				SST23_RS0101320			SST23_RS0101315	SST23_RS1000000111440	SST23_RS0101305	C
4253	EXW74_04430										B
BS26	JOA01_RS04580										D
BS27	JM961_05600										D
H35	KQ224_RS01520										B
SUT-7	SUT007_10200										B
SUT-286	L6410_RS04875	L6410_RS10595	L6410_RS10600	L6410_RS10605	L6410_RS10610	L6410_RS10585, L6410_RS10590	L6410_RS10580	L6410_RS10615	L6410_RS10620	L6410_RS10625	A
SUT-319	+*										D
SUT-328	+										D
SUT-380	SUT380_09340										D
SUT-503	SUT503_09340										D

* SUT-319 and SUT-328 haven’t be annotated by NCBI and do not have the locus tag

### CRISPR-Cas systems of *S. parasuis*

Three different type CRISPR-Cas system were found in *S. parasuis*, Type IC (repeats: GTCGCACCCTACACGGGTGCGTGGATTGAAAT), Type IIA (repeats: GTTTTAGAGCTGTGCTGTTTCGAATGGTTTCAAAAC) and Type IIC (repeats: GTTTTTGTACTCTCAAGATTTAAGTAACAGTAAAAC) (Fig. 5B). The average space counts of the *S. parasuis* CRISPR-Cas system was 45.7, and in isolates 4253, 127 spaces were found, suggesting that *S. parasuis* have a high frequency of interaction with other microbes or DNA fragments (Table 5).

**Table 5:** Prediction of CRISPR-Cas systems in *S. parasuis* genomes

Strains	CRISPR	Location	CRISPR type		Spacers Count
SUT-380	1	726,139–736,897	Type IC	cas3-cas5-cas8c-cas7-cas4-cas1-cas2	34
SUT-503	1	723,131–736,126	Type IC	cas3-cas5-cas8c-cas7-cas4-cas1-cas2	67
SUT-286	1	725,765–733,061	Type IIC	cas9-cas1-cas2	21
SUT-7	1	780,227–791,910	Type IIC	cas9-cas1-cas2	61
BS27	1	JAETXU010000002.1 (94678–102,604)	Type IIA	cas9-cas1-cas2-csn2	27
BS26	1	685,077–693,003	Type IIA	cas9-cas1-cas2-csn2	27
H35	0				
4253	1	SHGT01000014.1 (17035–33,827)	Type IC	cas3-cas5-cas8c-cas7-cas4-cas1-cas2	127
86–5192	1	ALLG01000027.1 (37949–47,662)	Type IC	cas3-cas5-cas8c-cas7-cas4-cas1-cas2	17
88–1861	0				
89–4109-1	1	ALLL01000005.1 (96237–107,761)	Type IC	cas3-cas5-cas8c-cas7-cas4-cas1-cas2	45
SUT-319	1	contig007 (68218–79,574)	Type IC	cas3-cas5-cas8c-cas7-cas4-cas1-cas2	43
SUT-328	1	contig007 (44096–55,452)	Type IC	cas3-cas5-cas8c-cas7-cas4-cas1-cas2	43
10–36,905	1	WNXH01000009.1 (26263–34,741)	Type IIA	cas9-cas1-cas2-csn2	36

### Potential virulence-associated genes identified by association analysis with the clinical condition of isolation source animals

Through scanning the genome of *S. parasuis*, all four classic virulence factors/markers of *S. suis* capsular polysaccharide (CPS), muramidase-released protein (MRP), suilysin (SLY) and extracellular factor (EF) are absent, suggesting that there may have other gene responsible for the virulence of *S. parasuis* (CPS has been considered as virulence marker because of the pathogenicity of isolates belongs to different serotypes is different, identify the serotype of *S. suis* isolates could speculate their virulence).

**Fig. 6:**
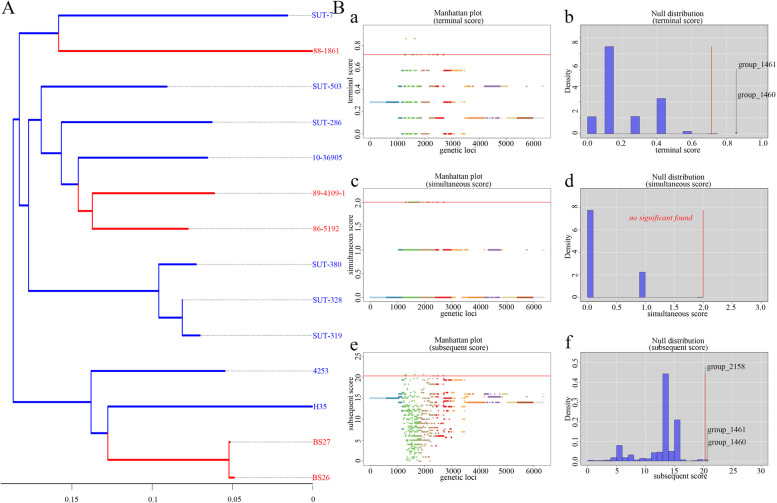
Identified genes associated with clinical condition of isolated source animals by treeWAS. **A** Phylogenetic tree based on the gene presence and absence. The isolates isolated from disease animals or humans are marked in red and from health animals or humans are marked in green; **B** The manhattan plots and null distributions of simulated association scores for (**a, b**) Terminal score, (**c, d**) Simultaneous score, (**e, f**) Subsequent score. A significance threshold is indicated in red, above which are associated genes

To further explore the potential virulence-associated genes of *S. parasuis*, the clinical condition of the isolation source animals was used as a dichotomous variable (disease and health), and combined analysis with the gene presence and absence matrix data generated by pan-genome analysis (Fig. 6). Three genes were identified as associated with this phenotype, and the functions of the coding-proteins of these genes were annotated (Table 6).

**Table 6:** Distribution of putative virulence associated genes

gene	product	10–36,905	4253	86–5192	88–1861	89–4109-1	BS26	BS27	H35	SUT-286	SUT-319	SUT-328	SUT-380	SUT-503	SUT-7
group_1460	type I restriction-modification system subunit R	/	/	SST18_RS0104060	SST34_RS0103785	SST23_RS0105740	JOA01_RS08475	JM961_06725	KQ224_RS08040	/	/	/	/	/	/
group_1461	type I restriction-modification system subunit M	/	/	SST18_RS0104035	SST34_RS0103755	SST23_RS0105715	JOA01_RS08495	JM961_06700	KQ224_RS08015	/	/	/	/	/	/
group_2158	glycosyltransferase family 1 protein	/	/	SST18_RS0105510		/	JOA01_RS06695	JM961_08320	/	/	/	/	/	/	/

## Discussion

*S. parasuis*, which initially belonged to *S. suis*, has been considered an opportunistic zoonotic pathogen. Exploring the genomic characteristics in whole genome level or compare some phenotypic determinants such as *cps* loci, ICE and pili cluster could improve our understanding of the bacteria’s molecular and evolutionary changes. In this study, we characterized the *S. parasuis* genomes obtained from public database, compared them with those of its close relative *S. suis*, and discussed their similarities and differences. However, given the large number of draft genomes were used in this study, which are incomplete and may contain contamination, the results may have some bias. *S. suis* is a zoonotic pathogen which can infect both humans and pigs world-wide [29–32]. However, although a diverse serotypes of *S. suis* could be detected in the upper respiratory tract of swine, most of them recovered from healthy animals. Considering that, *S. parasuis* may not only has potential as pathogen but also as commensal flora. Exploring the genomic characteristics of these bacteria could provide a novel understanding of the evolution of these bacteria.

Several studies have analysed the pan-genome of *S. suis* using the genomics method using different genome data. For example, Dong et al. analysed *S. suis* isolates from Europe, Asia, North America and South America to discuss the differences between isolates from different hosts, and the relationship between virulent ST1 European and epidemic ST7 Chinese isolates [33]. In this study, we found that the genomes of two human *S. parasuis* isolates were much shorter than those of the isolates from other sources. This finding is identical to previous studies in *S. suis*, which found that genome reduction is associated with bacterial pathogenicity, and the genome of human-associated *S. suis* isolates is much shorter than those from healthy or diseased pigs [33, 34]. However, the correlation of this phenomenon with genomic features is still unknown. Previous study speculated that genome reduction is driven by increased dependence on, or exploitation of, the host or bottlenecks associated with the increased transmission; however, genome reduction could be a useful marker of emerging and increasing pathogenicity [34]. Capsular polysaccharide is the determining component of the serotype of *Streptococci* [10]. CPS is also an essential virulence factor that plays a critical role in virulence formation and pathogenesis and is especially involved in the anti-phagocytosis mechanism of *Streptococci*, such as *S. suis* [35] and *S. pneumoniae* [36]. Therefore, it is also a vaccine target. The pneumococcal polysaccharide vaccine is one of the most successful microbial vaccines [37], meanwhile, the immunogenicity of CPS in *S. suis* is also being studied by Gottschalk, Segura and their colleagues [38, 39]. The CPS biosynthesis gene cluster drives the formation of streptococcal capsule of *Streptococci*. Previously Wang et al. aligned the *cps* locus difference between *S. parasuis* and *S. suis* and found that there were frequent interspecies horizontal transfers between the *cps* locus of *S. parasuis* and *S. suis*, and verified that both *S. parasuis* and *S. suis* synthesised their CPS using the WZX/WZY pathway [8]. In this study, the intraspecies difference of *S. parasuis* was explored, and we found 11 different *cps* types. There are more differences between different clades and more similarity inner the clades, however, it still needs to be proven by molecular analysis. It is worth noting that, although the *cps* locus is the determining cluster of *Streptococci* serotypes, it does not mean they belong to different serotypes if their *cps* locus are different. For example, the *cps* locus of virulent serotype 2 strains and avirulent serotype 2 strains of *S. suis* are also different, but they still belong to the same serotype [40]. Therefore, even though more than 10 *cps* types have been identified in *S. parasuis*, further tests such as serum agglutination tests are required to verify their differences.

Treatment with antibiotics is a simple and efficient way to treat bacterial infections. However, antibiotic misuse has resulted in the emergence of a number of multidrug-resistant microorganisms. Therefore, antimicrobial resistance (AMR) is an important public health concern. Resistance to tetracyclines, macrolides, and aminoglycosides was isolated at high rates in *S. suis*, particularly tetracycline and erythromycin, which are encoded by the resistance genes *erm(B)* and *tet(O)* [41, 42]. One of the crucial problems of antimicrobial resistance is the horizontal transfer of AMRGs. Some mobile structures of bacteria could be the medium to fulfil transmission, and plasmid and ICE are the most common structures. Plasmids containing AMRGs were already be reported in *S. suis* a long time ago [43, 44]. Recently, a plasmid harboring the oxazolidinone resistance gene *cfr(D)* in the *S. parasuis* genome was reported [45]. ICE is a kind of self-transmissible mobile genetic element that can horizontally transfer between prokaryotes, and was first reported in 2002 [46]. In *S. suis*, a series of ICEs containing various of AMRGs have been reported [47, 48]. In this study, 11 different ICEs were predicted in *S. parasuis*, which harboured AMRGs, by constructing a phylogenetic tree and aligning the sequence with *S. suis* ICE. The sequences of these ICEs from different species have a large range of similarities, and our findings indicate that these two pathogens may have the potential genetic exchange.

In many pathogenic bacteria, the pilus plays a pivotal role in host-pathogen interactions and the first colonization of specific host tissues [49]. Due to technological limitations, research on pili of Gram-positive bacteria has been relatively backward in the past century. In recent years, with the development of cell microbiology technology such as immunoelectron microscopy, an increasing number of more and more Gram-positive bacterial pili have been observed and proven to be associated with bacteria pathogenesis. Unlike Gram-negative bacteria, the surface molecules of Gram-positive bacteria are displayed on the cell wall because of the lack of an outer membrane [49]. Pili of many Gram-positive bacteria, such as *Corynebacterium diphtheriae* [50], *S. agalactiae* [51] and *S. pyogenes* [52] have already been identified as playing a very important role in the pathogenesis and involvement of adherence or virulence. In 2008, Takamatsu et al. identified four different pilus gene clusters in *S. suis*, according to the order of sortase, named as *srtBCD*, *srtE*, *srtF*, and *srtG*, based on the presence and absence of sortase, pilus and signal peptidase gene in these clusters. They separate *S. suis* to 12 genotypes [9], a subsequent study proved that the pili genotype is linked with MLST and virulence phenotype. Almost all of virulent serotype 2 *S. suis*, ST1 and ST7 are genotype A and avirulent serotype 2 *S. suis* ST28 is genotype B [53]. The role of these pili genes has been found to be associated with pathogenesis. The minor pili subunit, SBP1, is an adherence-associated protein [54], and the major pili subunit SBP2’ is important in *S. suis* virulence and cross-host transmission [55, 56]; In addition, sortases in these pilus gene clusters are reported to be essential for disease in pigs [57]. Furthermore, pili, known as antigens with good immunogenicity, have always been considered good subunit vaccines. Two major pili subunits of *srtBCD* and *srtF*, SBP2’ and SFP2, have been shown to have good immunogenicity and can provide immune protection to *S. suis* in mice model [58, 59]. Three different pilus gene clusters were identified in *S. parasuis*, one of which is homologous with *srtF* of *S. suis*, others are different from *S. suis*, and four different genotypes were found in *S. parasuis* based on the presence of pili clusters.

CRISPR, an acronym for Clustered Regularly Interspaced Short Palindromic Repeats, is an adaptive antiviral immunity system found in the DNA of many bacteria and archaea [60]. It was first found in 1987 in the genome of *Escherichia coli* isolate K12 [61] and is now broadly used in genome editing [62]. To date, a total of 2 classes, 6 types and 33 subtypes of CRISPR-Cas systems have been identified [63]. The spaces in different CRISPRs mostly correspond to fragments of similar lengths from foreign DNA, such as plasmids, bacteriophages, and mobile genetic elements [64]. Among the 14 *S. parasuis* isolate genomes, only two do not contain the CRISPR-Cas system, which is different from its close relative *S. suis*. CRISPR-Cas systems are rarely found in *S. suis*, scanning of the CRISPRCasdb, only 24 CRISPR-Cas systems of *S. suis* were found, considering that the number of genome sequences of *S. suis* in the public database is 1900+ (data from Genbank), which is a significant difference. Although, there are still some relatives between *S. suis* and *S. parasuis*, similar to *S. parasuis*, only Type IC, Type IIA, Type IIC were found in *S. suis*. This observation suggests that there may be potential interspecies exchanges of the CRISPR-Cas system among *Streptococcus* species.

One of the most important scientific concerns in bacteria pathogenesis is how virulence formed. The discovery of virulence factors and virulence-associated genes could help us dissect the pathogenic mechanisms of bacterial pathogens.. Two genes, group_1460 (*hsdR*) and group_1461 (*hsdM*), belong to the type I restriction-modification system, which is broadly present in *Streptococci* and has already been well studied. This system regulates gene expression and virulence of pneumococci as a random six-phase switch epigenetics [65]. Similar functions have also been reported in *S. suis*; this phase-variable methyltransferases system may also be involved in the virulence formation of *S. suis* [66, 67]. Considering that the complete type I restriction-modification system in *S. pneumoniae* and *S. suis* contain at least three genes, *hsdS*, *hsdR* and *hsdM*, and only *hsdR* and *hsdM* were identified, we scanned the genomes of these *hsdR* and *hsdM* positive *S. parasuis* isolates, all of which contain a *hsdS* gene adjoining with *hsdR* and *hsdM*. However, further alignment of the sequence of these *hsdS* genes found that the similarity of these *hsdS* sequences is lower than 50%; thus, the pan-genome analysis has not grouped them into one group, which is why *hsdS* has not been identified to be associated with clinical conditions. Further experiments are required to determine whether these *hsdS* gene play different roles in pathogenesis of *S. parasuis*. Group_2158, which encodes a glycosyltransferase family protein, belongs to the middle section of *S. parasuis cps* locus. As we discussed in Section 3.4, CPS is an essential virulence factor of *S. suis* and other *Streptococci*. Taken together, we found three potential virulence-associated genes of *S. parasuis* in this study and their role in *S. parasuis* could be evaluated by further research.

## Conclusion

We examined all genomes of *S. parasuis* from public database to explore their main genetic features and differences from those of its close relative *S. suis*. Our data provide novel insights into the interspecies and intraspecies genetic characteristics of *S. parasuis* through pan-genome phylogeny, analysis of capsular polysaccharide loci, migration potential antimicrobial resistance genes, pilus gene clusters, CRISPR-Cas systems, and virulence-associated genes, which can be useful for further study of this species, such as serotyping, diagnostics, vaccine development, and study of the pathogenesis mechanism. In addition, we propose to consider the horizontal gene exchange potential between this species and *S. suis*.

## Supplementary Information

**Additional file 1.** Quality assessment of the draft genomes used in this study

**Additional file 2. **Location of the capsular polysaccharide biosynthesis loci of *S. parasuis*

**Additional file 3. **Details of ICEs of *Streptococcus suis* from ICEberg

**Additional file 4. **Details of pili cluster of *Streptococcus suis* used in this study

**Additional file 5. **Heatmap based on (**A**) average nucleotide identity (ANI) and (**B**) tetranucleotide frequencies (Tetra) of 14 *S. parasuis* and isolate 2843; **C** gene presence and absence matrix of 14 *S. parasuis* and isolate 2843, isolate 2843 is marked by a red rectangle.

**Additional file 6.** Mauve comparison diagrams of the BS26, SUT-286, SUT-380, SUT-503 and SUT-7 genomes.

**Additional file 7. **Phylogenetic analysis the MLST of 14 *S. parasuis* isolates. The confidence values were obtained from 1000 replications.

**Additional file 8.** Pie chart of the breakdown of genes and the number of isolates in which they were present.

**Additional file 9. **Colinear analysis of the capsular polysaccharide (CPS) biosynthesis locus of *S. parasuis* using Mauve.

## Data Availability

The datasets generated and/or analyzed during the current study are available in the GenBank repository, the Accession Number of all genome sequences used in this study is AP024277.1, AP024280.1, AP024276.1, AP024275.1, JAETXU000000000.1, CP069079.1, CP076721.1, SHGT00000000.1, ALLG00000000.1, ALLW00000000.1, ALLL00000000.1, DRX016753, DRX016754, WNXH00000000.1 and POIG00000000.1.
