# Supplementary material for: Genomic characterization of Streptococcus parasuis, a close relative of Streptococcus suis and also a potential opportunistic zoonotic pathogen
Source: BMC Genomics. 2022 Jun 25;23:469. doi: 10.1186/s12864-022-08710-6 (PMC9233858; doi:10.1186/s12864-022-08710-6)
Supplement: Supplementary file 1 — Additional file 1. Quality assessment of the draft genomes used in this study [file 12864_2022_8710_MOESM1_ESM.docx]

| Additional file 1. Quality assessment of the draft genomes used in this study | | | | | | | | | |
| --- | --- | --- | --- | --- | --- | --- | --- | --- | --- |
| Assembly | BS27 | 4253 | 10_36905 | 86_5192 | 88_1861 | 89_4109_1 | 2843 | SUT_319 | SUT_328 |
| # contigs (>= 0 bp) | 25 | 106 | 36 | 55 | 102 | 76 | 488 | 53 | 49 |
| # contigs (>= 1000 bp) | 22 | 106 | 30 | 52 | 92 | 71 | 78 | 34 | 34 |
| # contigs (>= 5000 bp) | 14 | 81 | 25 | 39 | 71 | 51 | 52 | 29 | 29 |
| # contigs (>= 10000 bp) | 12 | 59 | 24 | 36 | 60 | 43 | 46 | 23 | 23 |
| # contigs (>= 25000 bp) | 9 | 22 | 23 | 24 | 38 | 31 | 29 | 20 | 20 |
| # contigs (>= 50000 bp) | 7 | 5 | 18 | 17 | 12 | 14 | 12 | 17 | 18 |
| Total length (>= 0 bp) | 1909795 | 1881656 | 2148541 | 2110166 | 2272254 | 2176728 | 2267031 | 2128604 | 2086527 |
| Total length (>= 1000 bp) | 1908138 | 1881656 | 2145911 | 2109324 | 2266428 | 2173671 | 2133271 | 2118751 | 2078036 |
| Total length (>= 5000 bp) | 1889628 | 1818135 | 2137736 | 2081081 | 2212327 | 2134632 | 2070650 | 2105045 | 2064136 |
| Total length (>= 10000 bp) | 1877812 | 1657114 | 2132278 | 2062771 | 2135475 | 2076322 | 2025770 | 2063144 | 2019370 |
| Total length (>= 25000 bp) | 1827395 | 1001237 | 2115034 | 1864693 | 1787133 | 1866706 | 1740101 | 1999297 | 1954989 |
| Total length (>= 50000 bp) | 1751354 | 395498 | 1967240 | 1598779 | 951387 | 1297186 | 1109439 | 1895068 | 1871213 |
| # contigs | 24 | 106 | 32 | 52 | 98 | 74 | 98 | 42 | 41 |
| Largest contig | 541704 | 125206 | 264006 | 202666 | 165407 | 198824 | 134547 | 250294 | 166094 |
| Total length | 1909460 | 1881656 | 2147346 | 2109324 | 2270792 | 2176041 | 2146482 | 2125082 | 2083551 |
| GC (%) | 39.69 | 39.9 | 39.78 | 39.94 | 39.68 | 39.88 | 43.49 | 39.71 | 39.67 |
| N50 | 453348 | 25681 | 119199 | 86346 | 35704 | 64745 | 58042 | 123669 | 123669 |
| N75 | 138725 | 18061 | 66641 | 56156 | 26790 | 30772 | 32505 | 74405 | 74396 |
| L50 | 2 | 20 | 7 | 9 | 17 | 11 | 12 | 7 | 7 |
| L75 | 5 | 42 | 13 | 17 | 35 | 23 | 25 | 12 | 13 |
| Marker Lineage | o__Lactobacillales | o__Lactobacillales | o__Lactobacillales | o__Lactobacillales | o__Lactobacillales | o__Lactobacillales | o__Lactobacillales | o__Lactobacillales | o__Lactobacillales |
| # Genomes | 293 | 293 | 293 | 293 | 293 | 293 | 293 | 293 | 293 |
| # Markers | 475 | 475 | 475 | 475 | 475 | 475 | 475 | 475 | 475 |
| # Marker Sets | 267 | 267 | 267 | 267 | 267 | 267 | 267 | 267 | 267 |
| 0 | 0 | 0 | 0 | 0 | 1 | 0 | 1 | 0 | 0 |
| 1 | 474 | 475 | 475 | 475 | 473 | 475 | 474 | 475 | 475 |
| 2 | 1 | 0 | 0 | 0 | 1 | 0 | 0 | 0 | 0 |
| 3 | 0 | 0 | 0 | 0 | 0 | 0 | 0 | 0 | 0 |
| 4 | 0 | 0 | 0 | 0 | 0 | 0 | 0 | 0 | 0 |
| 5+ | 0 | 0 | 0 | 0 | 0 | 0 | 0 | 0 | 0 |
| Completeness | 100 | 100 | 100 | 100 | 99.81 | 100 | 99.63 | 100 | 100 |
| Contamination | 0.37 | 0 | 0 | 0 | 0.37 | 0 | 0 | 0 | 0 |
