# Supplementary material for: Genomic characterization of Streptococcus parasuis, a close relative of Streptococcus suis and also a potential opportunistic zoonotic pathogen
Source: BMC Genomics. 2022 Jun 25;23:469. doi: 10.1186/s12864-022-08710-6 (PMC9233858; doi:10.1186/s12864-022-08710-6)
Supplement: Supplementary file 2 — Additional file 2. Location of the capsular polysaccharide biosynthesis loci of S. parasuis [file 12864_2022_8710_MOESM2_ESM.docx]

| Additional file 2. Location of the capsular polysaccharide biosynthesis loci of *S. parasuis* | |
| --- | --- |
| Strains | Location |
| SUT-380 | 1454159 - 1482339 |
| SUT-503 | 1412294 - 1461359 |
| SUT-286 | 1445127 - 1480295 |
| SUT-7 | 1521746 - 1557853 |
| BS27 | JAETXU010000001.1 (272526 - 303128) |
| BS26 | 1298755 - 1329355 |
| H35 | 1910139 - 1954893 |
| 4253 | SHGT01000017.1 (25289 - end), SHGT01000040.1, SHGT01000022.1 (start - 8493) |
| 86-5192 | ALLG01000019.1 (25511 - 60928) |
| 88-1861 | ALLW01000087.1 (start - 19304), ALLW01000098.1 (start - 23016) |
| 89-4109-1 | ALLL01000060.1 (115926 - end), ALLL01000061.1, ALLL01000062.1, ALLL01000063.1, ALLL01000037.1 (start - 22285) |
| SUT-319 | contig006 (36329 - 64652) |
| SUT-328 | contig005 (36325 - 64648) |
| 10-36905 | WNXH01000003.1 (25911 - 57861) |
