# Supplementary material for: Genomic characterization of Streptococcus parasuis, a close relative of Streptococcus suis and also a potential opportunistic zoonotic pathogen
Source: BMC Genomics. 2022 Jun 25;23:469. doi: 10.1186/s12864-022-08710-6 (PMC9233858; doi:10.1186/s12864-022-08710-6)
Supplement: Supplementary file 3 — Additional file 3. Details of ICEs of Streptococcus suis from ICEberg [file 12864_2022_8710_MOESM3_ESM.docx]

| Additional file 3. Details of ICEs of *Streptococcus suis* from ICEberg | | | | |
| --- | --- | --- | --- | --- |
| ICEberg ID | Name | Size (bp) | GC content (%) | Nucleotide Sequence |
| 829 | ICESsu05SC260 | 88874 | 36.81 | KX077888 |
| 311 | ICESsu05ZYH33-1 | 88851 | 38.79 | CP000407 |
| 445 | ICESsu32457 | 54879 | 38.89 | FR823304 |
| 313 | ICESsu98HAH33-1 | 18031 | 38.79 | CP000408 |
| 232 | ICESsuBM407-1 | 75717 | 37.81 | FM252032 |
| 232 | ICESsuBM407-2 | 80320 | 37.11 | FM252032 |
| 833 | ICESsuJH1301 | 81649 | 37.24 | KX077887 |
| 834 | ICESsuJH1308-1 | 79260 | 37.17 | KX077886 |
| 835 | ICESsuJH1308-2 | 75677 | 37.45 | KX077884 |
| 836 | ICESsuLP081102 | 64515 | 38.25 | KX077885 |
| 234 | ICESsuSC84 | 89165 | 36.84 | FM252031 |
| 837 | ICESsuNC28 | 29661 | 34.14 | KU215704 |
| 641 | ICESsuTZ080501 | 125779 | 39.05 | KX077897 |
| 642 | ICESsuYY060816 | 125757 | 39.06 | KX077898 |
| 840 | ICESsuZJ20091101-1 | 69397 | 38.98 | KX077882 |
| 841 | ICESsuZJ20091101-2 | 112763 | 38.38 | KX077883 |
