# Supplementary material for: Genomic characterization of Streptococcus parasuis, a close relative of Streptococcus suis and also a potential opportunistic zoonotic pathogen
Source: BMC Genomics. 2022 Jun 25;23:469. doi: 10.1186/s12864-022-08710-6 (PMC9233858; doi:10.1186/s12864-022-08710-6)
Supplement: Supplementary file 4 — Additional file 4. Details of pili cluster of Streptococcus suis used in this study [file 12864_2022_8710_MOESM4_ESM.docx]

| Additional file 4. Details of pili cluster of *Streptococcus suis* used in this study | | | | |
| --- | --- | --- | --- | --- |
| Pili cluster | gene | origin strain | accession | locus tag |
|  | srtA | P1/7 | NC_012925.1 | SSU_RS04715 |
| srtBCD cluster | srtB | ZY05719 | NZ_CP007497.1 | ZY05719_RS09895 |
|  | srtC |  |  | ZY05719_RS09895 |
|  | srtD |  |  | ZY05719_RS09890 |
|  | sbp1 |  |  | ZY05719_RS09905 |
|  | sbp2' |  |  | ZY05719_RS10950 |
|  | sbp2'' |  |  | ZY05719_RS10945 |
|  | sbp3 |  |  | ZY05719_RS09920 |
|  | sbp4 |  |  | ZY05719_RS09925 |
| srtE cluster | srtE | NCTC10237 | NZ_LR594043.1 | FGL16_RS02765 |
|  | sep1 |  |  | FGL16_RS02760 |
|  | sep2 |  |  | FGL16_RS02755 |
|  | sipE |  |  | FGL16_RS02750 |
| srtF cluster | srtF | P1/7 | NC_012925.1 | SSU_RS02350 |
|  | sfp1 |  |  | SSU_RS02345 |
|  | sfp2 |  |  | SSU_RS02340 |
|  | sipF |  |  | SSU_RS02330 |
| srtG cluster | srtG | 05HAS68 | NZ_CP002007.2 | HAS68_RS07955 |
|  | sgp1 |  |  | HAS68_RS07960 |
|  | sgp2 |  |  | HAS68_RS07965 |
