# Supplementary material for: Genomic characterization of Streptococcus parasuis, a close relative of Streptococcus suis and also a potential opportunistic zoonotic pathogen
Source: BMC Genomics. 2022 Jun 25;23:469. doi: 10.1186/s12864-022-08710-6 (PMC9233858; doi:10.1186/s12864-022-08710-6)
Supplement: Supplementary file 5 — Additional file 5. Heatmap based on (A) average nucleotide identity (ANI) and (B) tetranucleotide frequencies (Tetra) of 14 S. parasuis and isolate 2843; C gene presence and absence matrix of 14 S. parasuis and isolate 2843, isolate 2843 is marked by a red rectangle. [file 12864_2022_8710_MOESM5_ESM.docx]

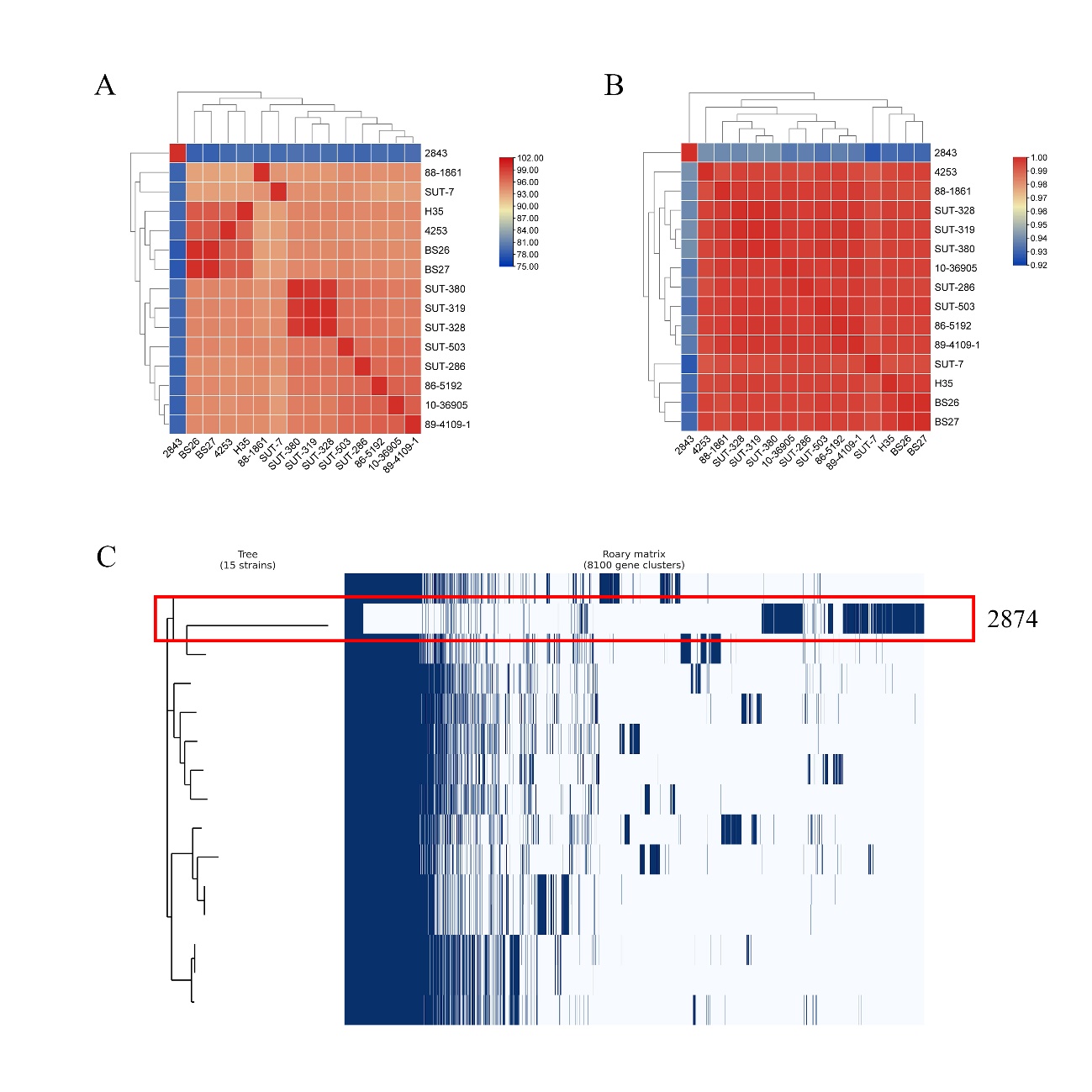
 Additional file 5: Heatmap based on A) average nucleotide identity (ANI) and B) tetranucleotide frequencies (Tetra) of 14 *S. parasuis* and isolate 2843; C) gene presence and absence matrix of 14 *S. parasuis* and isolate 2843, isolate 2843 is marked by a red rectangle.
