# Supplementary figures and images for: Genomic characterization of Streptococcus parasuis, a close relative of Streptococcus suis and also a potential opportunistic zoonotic pathogen
Source: BMC Genomics. 2022 Jun 25;23:469. doi: 10.1186/s12864-022-08710-6 (PMC9233858; doi:10.1186/s12864-022-08710-6)

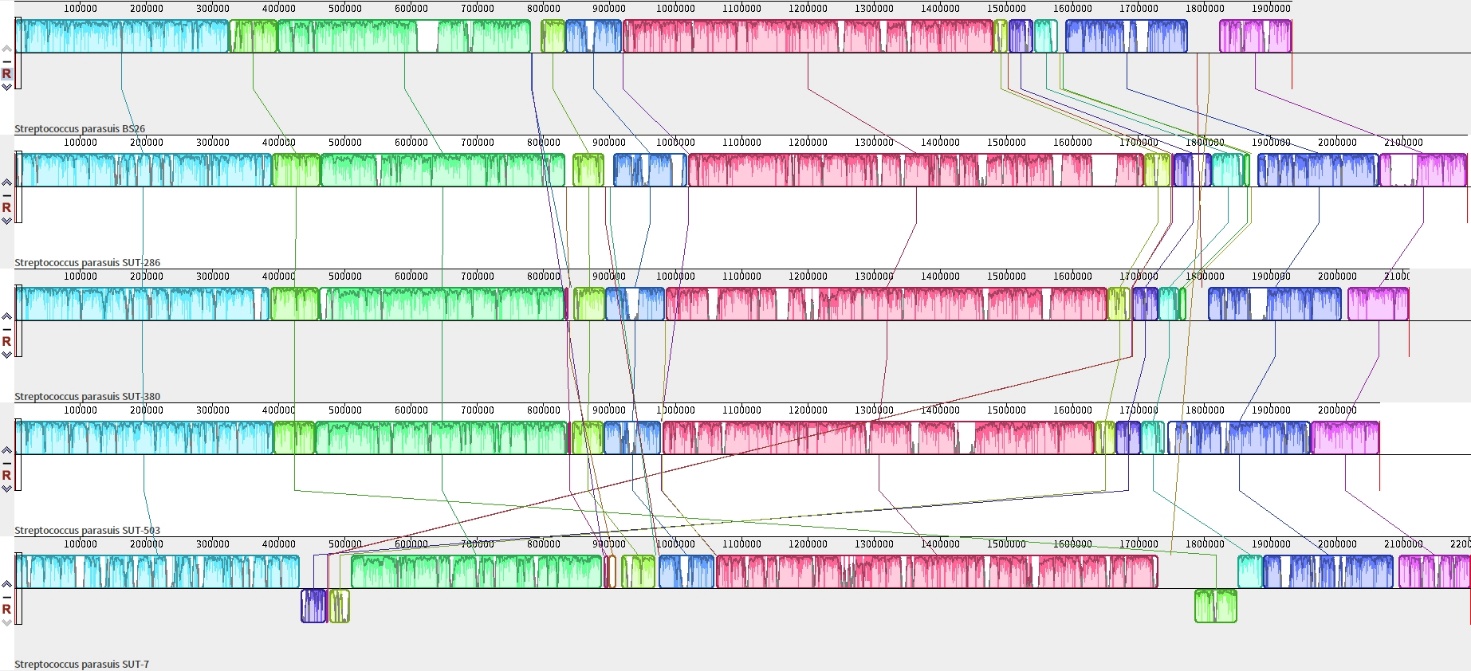


Additional file 6: Mauve comparison diagrams of the BS26, SUT-286, SUT-380, SUT-503 and SUT-7 genomes.

Supplement: Supplementary file 6 — Additional file 6. Mauve comparison diagrams of the BS26, SUT-286, SUT-380, SUT-503 and SUT-7 genomes. [file 12864_2022_8710_MOESM6_ESM.docx]
