# Supplementary material for: Genomic characterization of Streptococcus parasuis, a close relative of Streptococcus suis and also a potential opportunistic zoonotic pathogen
Source: BMC Genomics. 2022 Jun 25;23:469. doi: 10.1186/s12864-022-08710-6 (PMC9233858; doi:10.1186/s12864-022-08710-6)
Supplement: Supplementary file 7 — Additional file 7. Phylogenetic analysis the MLST of 14 S. parasuis isolates. The confidence values were obtained from 1000 replications. [file 12864_2022_8710_MOESM7_ESM.docx]

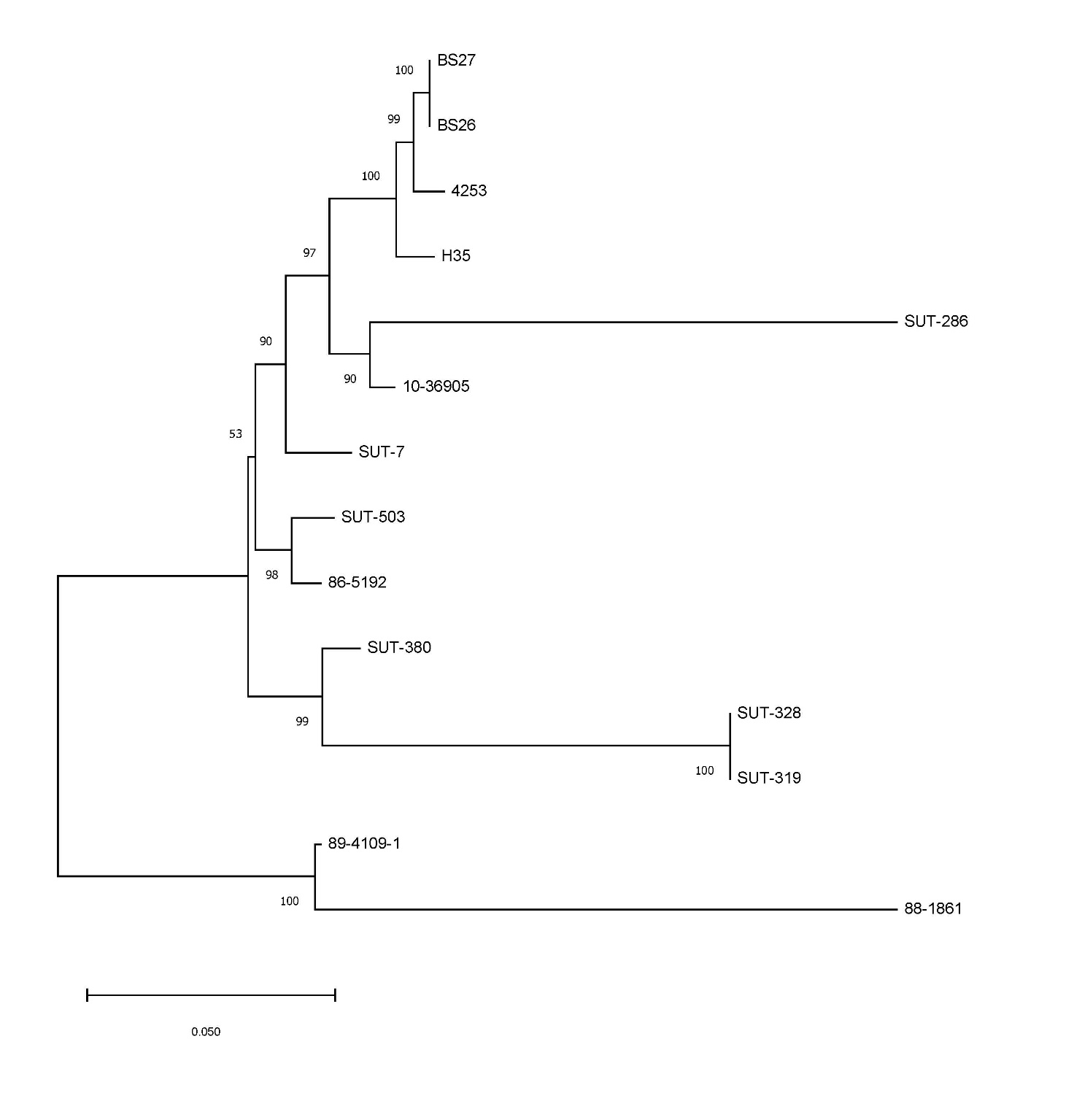


Additional file 7: Phylogenetic analysis the MLST of 14 *S. parasuis* isolates. The confidence values were obtained from 1000 replications.
