# Supplementary material for: Genomic characterization of Streptococcus parasuis, a close relative of Streptococcus suis and also a potential opportunistic zoonotic pathogen
Source: BMC Genomics. 2022 Jun 25;23:469. doi: 10.1186/s12864-022-08710-6 (PMC9233858; doi:10.1186/s12864-022-08710-6)
Supplement: Supplementary file 8 — Additional file 8. Pie chart of the breakdown of genes and the number of isolates in which they were present. [file 12864_2022_8710_MOESM8_ESM.docx]

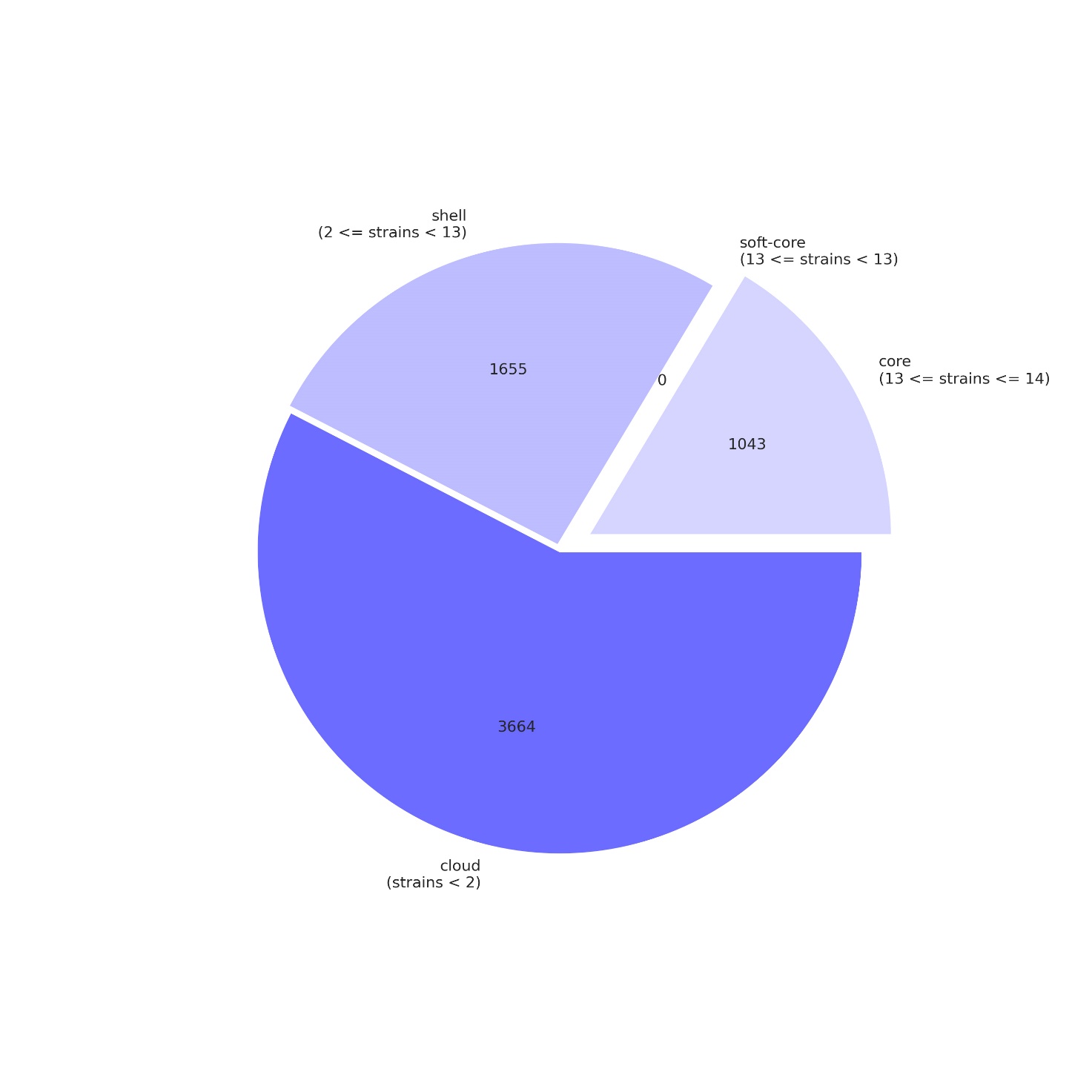


Additional file 8: Pie chart of the breakdown of genes and the number of isolates in which they were present.
