# Supplementary material for: Genomic characterization of Streptococcus parasuis, a close relative of Streptococcus suis and also a potential opportunistic zoonotic pathogen
Source: BMC Genomics. 2022 Jun 25;23:469. doi: 10.1186/s12864-022-08710-6 (PMC9233858; doi:10.1186/s12864-022-08710-6)
Supplement: Supplementary file 9 — Additional file 9. Colinear analysis of the capsular polysaccharide (CPS) biosynthesis locus of S. parasuis using Mauve. [file 12864_2022_8710_MOESM9_ESM.docx]

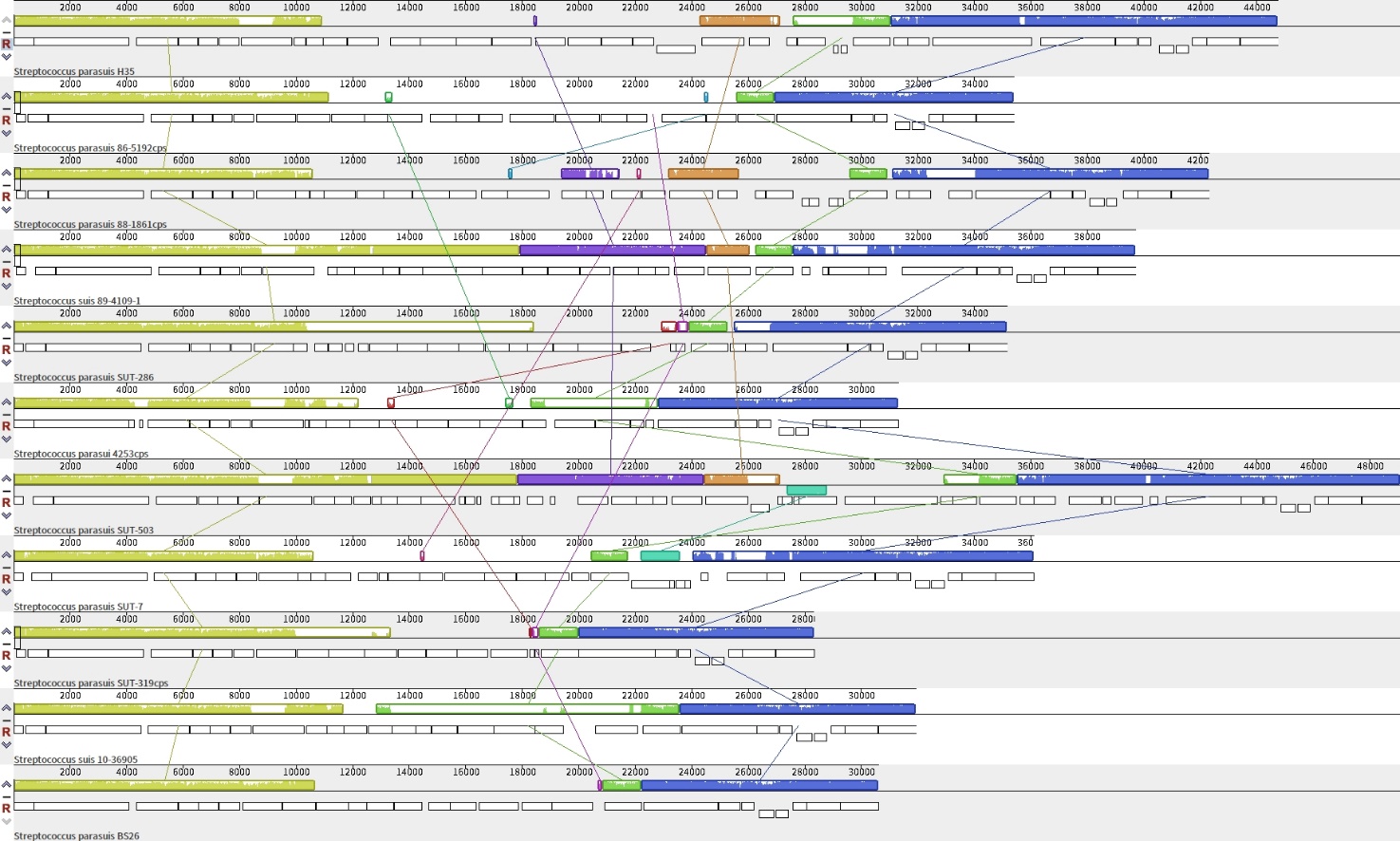


Additional file 9: Colinear analysis of the capsular polysaccharide (CPS) biosynthesis locus of *S. parasuis* using Mauve.
